# Dissemination of a single ST11 clone of OXA-48-producing *Klebsiella pneumoniae* within a large polyclonal hospital outbreak determined by genomic sequencing

**DOI:** 10.1099/mgen.0.000808

**Published:** 2022-04-08

**Authors:** Fernando Lázaro-Perona, Elias Dahdouh, Alma Sotillo, Verónica Pérez-Blanco, Jennifer Villa, Esther Viedma, Guillermo Ruiz-Carrascoso, Jesús Mingorance

**Affiliations:** ^1^​ Servicio de Microbiología, Hospital Universitario La Paz, IdiPAZ, Paseo de La Castellana 261, 28046 Madrid, Spain; ^2^​ Servicio de Medicina Preventiva, Hospital Universitario La Paz, IdiPAZ, Paseo de La Castellana 261, 28046 Madrid, Spain; ^3^​ Servicio de Microbiología, Hospital Universitario 12 de Octubre, Imas12, Avenida de Córdoba sn, Madrid 28041, Spain

**Keywords:** OXA-48-producing *Klebsiella pneumoniae*, hospital outbreak, OXA-48, ST11, carbapenemases, genomic analysis, phylodynamics

## Abstract

The population structure of a set of OXA-48-producing *Klebsiella pneumoniae* isolates belonging to sequence type 11 (ST11 Kp-OXA) and obtained from two hospitals in Madrid in the period from 2012 to 2015 was studied by genome sequencing. Overall, 97 ST11 Kp-OXA isolates were sequenced and their population structure and demography were studied by Bayesian phylodynamic analysis using core-genome SNVs. In total, 92 isolates were from Hospital La Paz, 57 of them from two selected units. The remaining five isolates were from different units of Hospital Doce de Octubre. Altogether, 96 out of the 97 ST11 Kp-OXA isolates could be ascribed to a single lineage that evolved into three sublineages. Demographic inference showed an expansion of the ST11 Kp-OXA in the first half of 2013 in agreement with the registered incidences. Dated phylogeny showed transmission clusters within hospital wards, between wards and between hospitals. The ST11 Kp-OXA outbreak in Hospital La Paz was largely due to the expansion of a single clone that was transmitted between different units and to Hospital Doce de Octubre. This clone diverged into three sub-lineages and spread out following a mixed mode of neutral core-genome evolution with some features of antibiotic selection, frequent large deletions and plasmid loss and gain events.

## Data Summary

**Impact Statement**

OXA-48-producing *Klebsiella pneumoniae* have been responsible of numerous hospital outbreaks in the Mediterranean area during the last decade. We have sequenced the genomes of 97 isolates belonging to a major lineage, ST11. The study included 92 isolates from different wards of Hospital Universitario La Paz and five isolates from Hospital Universitario Doce de Octubre, also in Madrid. Sequence analysis showed that all but one of the isolates were closely related and consistent with the expansion of a single lineage. Bayesian phylodynamic analysis including the isolation dates inferred a time-scaled phylogeny that suggests local transmission and expansion within units but also transmission between units and even between hospitals.

All the genome sequence data have been deposited in NCBI GenBank under Bioproject ID PRJNA757198. The assembled and annotated genome of isolate Kp11-81, which is used as the reference genome throughout the project, has been deposited in the same Bioproject with accession numbers: CP082154, CP082155, CP082156 and CP082157. Relevant metadata on sample isolation dates and sites are presented in Table S3 (available in the online version of this article). Supplementary Material for this article can be found on Figshare at: https://doi.org/10.6084/m9.figshare.17705984 [1].

## Introduction

OXA-48-producing *Klebsiella pneumoniae* (Kp-OXA) are multidrug-resistant pathogens that have spread through the Mediterranean region during the last decade [2]. In Hospital Universitario La Paz (HULP), the first cases, detected in 2010 [3], belonged to sequence type 405 (ST405) and this was the most frequent type during the first 3 years of the outbreak. Sporadic ST11 Kp-OXA isolates were found in 2011, their frequency increased in 2012 and they outcompeted ST405, becoming the most common sequence type from mid-2013 to 2015 [4]. Later, ST11 was replaced by other types (e.g. ST15, ST307) in a polyclonal distribution with no single dominant type.

ST11 is one of the major *K. pneumoniae* pathogenic clones, has a global distribution and has been often involved in hospital outbreaks of ESBL- and carbapenemase-producing *K. pneumoniae* [5, 6]. Carbapenem-resistant ST11 *K. pneumoniae* may harbour different carbapenemase genes, including *bla*_KPC_, *bla*_NDM_, *bla*_VIM_ and *bla*_OXA-48_ [5, 7]. ST11 is closely related to ST258 [8] and together they constitute the bulk of the globally distributed clonal complex 258 (CC258) [7]. ST11 genomes can be grouped into three clades: clade 1 has a worldwide distribution and clades 2 and 3 are restricted to China [6, 9]. Pan-genome analysis of 364 ST11 genomes has shown a large diversity, an open pan-genome and more than 19 000 genes. Clade 1 is the most diverse and includes several capsular types. Clades 2 and 3 have capsule types KL47 and KL64, respectively, and fewer unique genes and core-genome single nucleotide variants (SNVs) [5].

In Spain, ST11 Kp-OXA have been found in complex polyclonal hospital outbreaks [3, 10, 11], in outpatients [4], environmental samples [12] and even colonizing wild avian species [13]. This broad distribution and the polyclonal nature of most Kp-OXA outbreaks raised the question about the clonality of ST11 Kp-OXA outbreaks and whether they derive from the expansion of a single clone or from independent *bla*_OXA-48_ plasmid acquisition events by different ST11 lineages.

## Methods

### Samples

In total, 97 isolates of OXA-48-producing *K. pneumoniae* belonging to ST11 (Kp-OXA-ST11) were selected among those obtained from clinical and surveillance samples (rectal swabs) following the routine procedures of the Clinical Microbiology Departments of HULP and Hospital Universitario Doce de Octubre (H12O). One isolate per patient was selected. Then, 35 isolates obtained between 2012 and 2014 were selected from 18 different HULP wards, five isolates were selected among those obtained in H12O between 2013 and 2015, 32 were selected among those obtained from a HULP surgical ward (Vascular Surgery) between 2012 and 2015 and 25 from a HULP medical ward (Nephrology) in the same time period. No other selection criteria were applied. Altogether, 66 isolates were obtained from rectal swabs, 13 from blood cultures, seven from urine, five from respiratory samples, two from abdominal abscesses, two from surgical wounds and two from skin ulcers. Species identification was done with the MALDI Biotyper system (BrukerDaltonik, Bremen, Germany). Sequence type identification was done by clone-specific PCR [14] in HULP, and by Multi-Locus Sequence Typing (MLST) [15] in H12O. Antibiotic susceptibility testing was done with the Wider (Francisco Soria Melguizo, Madrid, Spain) and MicroScan WalkAway systems (Beckman Coulter, Brea, CA, USA) and interpretation was done according to EUCAST V3.1. The study was approved by the ethics committee of Hospital Universitario La Paz.

### Genome sequencing

Whole-genome sequencing was done by Eurofins Genomics (Ebersberg, Germany) in 2×125 sequencing mode in a HiSeq 2500 (Illumina, San Diego, CA, USA) using HiSeq SBS Kit v4 (Illumina, San Diego, CA, USA).

The oldest isolate (Kp11-81, February 2012) was selected as the reference isolate and was re-sequenced using a MinION MkI sequencer (Oxford Nanopore Technology, Oxford, UK) with the Nanopore rapid library preparation kit (Oxford Nanopore Technology, Oxford, UK) in an R9 flowcell (Oxford Nanopore Technology, Oxford, UK). Isolate Kp11-14 was re-sequenced using the same sequencer and library preparation kit and a Flongle flow cell (Oxford Nanopore Technology, Oxford, UK).

All the sequences have been deposited in the GenBank database under Bioproject accession number PRJNA757198. The genome of Kp11-81 has been deposited in the same Bioproject with accession numbers: CP082154, CP082155, CP082156 and CP082157, and the draft genome of Kp11-14 has been deposited with accession number: JAKNSM000000000.

### Bioinformatic sequence analyses

*De novo* assembly of the reference sequence (Kp11-81) was done with Unicycler (v0.4.4) [16] using the two sets of reads (short Illumina reads and long Nanopore reads) and the closed genome was annotated using the NCBI Prokaryotic Genome Annotation Pipeline [17]. Isolate Kp11-14 was assembled with Unicycler (v.0.4.9) using long and short reads.

The other genomes were assembled and compared with the reference genome using the *Nullarbor* pipeline (v1.30) [18]. *Nullarbor*-assembled genomes have been deposited together with the raw reads in GenBank (Bioproject PRJNA757198). The genomes were also analysed by mapping the paired reads to the reference sequence using Geneious R10.2 (Biomatters, Auckland, New Zealand) and large deletions and duplications were detected by visual inspection of coverage maps. Plasmids and antibiotic resistance genes were searched locally using blast [19] (whithin the Geneious package) and the PlasmidFinder and ResFinder databases (downloaded from http://www.genomicepidemiology.org/, PlasmidFinder database updated 20 February 2017 and ResFinder database updated 2 November 2016). Sequence types were confirmed by *Nullarbor* for all the isolates and using MLST 2.0 (Center for Genomic Epidemiology, https://cge.cbs.dtu.dk/services/MLST/) [20, 21] for the whole Kp11-81 assembled genome and the raw reads of Kp11-38. Capsule typing was done using Kaptive’s web interface (https://kaptive-web.erc.monash.edu/) [22].

Dated phylogeny was reconstructed using Bayesian inference with the Beast 2.6.3 package using the SNVs of the core genome with ascertainment correction [23]. Sampling date was expressed in years until the date of the most recent isolate. A relaxed lognormal clock model and a coalescent bayesian skyline prior were used. MCMC chains were run for 100 million steps with sampling every 10000 steps. Convergence was evaluated using Tracer v1.7.1 [24]. Trees were summarized as maximum clade credibility trees using TreeAnnotator v1.8.4 and then visualized with FigTree v1.4.4 [25].

## Results

### Sequencing of ST11 Kp-OXA genomes

In total, 97 isolates of ST11 Kp-OXA were selected for sequencing. Overall, 92 from Hospital Universitario La Paz, a third-level hospital in the northern area of the city of Madrid. Then, 35 had been obtained from 18 different hospital wards between 2012 and 2014, 32 from a surgical ward (Vascular Surgery) between 2012 and 2015 and 25 from a medical ward (Nephrology) in the same time period. These two wards had persistent outbreaks of ST11 Kp-OXA. Five isolates were obtained from Hospital Universitario Doce de Octubre, a third-level hospital in the southern part of Madrid. They had been isolated in different wards between 2013 and 2015. DNA was purified from these isolates and sequenced in 125×2 paired-ends mode (Table S1).

The oldest isolate within this set, Kp11-81, was selected for long read sequencing with Oxford Nanopore Technonologies’ MinION system. The long and short reads were assembled together to generate four scaffolds: the chromosome (5327910 bp) and three plasmids: a 220333 bp IncFIB plasmid (p220F), a 66808 bp IncR plasmid (p66R) and a 65626 bp IncL plasmid (p65L). The closed genome of Kp11-81 was used as the reference sequence.

Annotation of Kp11-81 sequence by the NCBI Prokaryotic Genome Annotation Pipeline identified 5382 coding sequences in the chromosome, 12 non-coding RNA genes, 25 rRNA genes, 86 tRNA genes and one tmRNA gene. Capsule locus typing identified the KL24 capsular locus that encodes capsular serotype K24. Several antibiotic resistance mutations and genes were detected (Table 1). The *bla*_OXA-48_ gene was located in plasmid p65L, which was identical to several pOXA-48 plasmids deposited in the public databases [26, 27]. Two *bla_CTX-M-15_* genes were detected, one in the chromosome and the other in the p66R plasmid. This one also contained the *bla*_TEM_ and *bla*_OXA-1_ genes (Table 1).

**Table 1.** Antibiotic resistance genes and mutations identified by ResFinder in the genome of Kp11-81. Efflux pumps are not included. This isolate was resistant to all beta-lactams tested except cefoxitin (MIC 16 mg l^−1^), imipenem (MIC 2 mg l^−1^) and meropenem (MIC 1 mg l^−1^); it was resistant also to fluoroquinolones, nitrofurantoin, fosfomycin, trimethoprim/sulfamethoxazole, gentamicin and tobramycin, and susceptible to amikacin (MIC ≤8 mg l^−1^); tigecycline (MIC ≤1 mg l^−1^) and colistin (MIC ≤2 mg l^−1^)

Scaffold	Resistance gene/mutation	Position in scaffold	Resistance to
chromosome	*catA1*	165001.165660	Phenicols
	*gyrA* S83F	233042.230409	Fluoroquinolones
	*parC* S80I	1052421.1054679	Fluoroquinolones
	*uhpT* E350Q	1722024.1723415	Fosfomycin
	*fosA*	2446264.2446675	Fosfomycin
	*blaSHV-11*	4366286.4367146	Beta-lactams
	*blaCTX-M-15*	4932637.4933512	Beta-lactams
p220F	*aph(3')-Ia*	147942.148757	Aminoglycosides
	*mph(A*)	149734.150639	Macrolides
	*sul1*	155950.156789	Sulphonamides
	*qacE*	156849.157130	Quaternary ammonium Disinfectants
	*aadA2*	157294.158085	Aminoglycosides
	*dfrA12*	158493.158990	Trimethoprim
p66R	*aac(6')-Ib-cr*	30204.30803	Fluoroquinolones and aminoglycosides
	*blaOXA-1*	30934.31764	Beta-lactams
	*catB3*	31902.32343	Phenicols
	*qnrB1*	37568.38212	Quinolones
	*aac(3)-IIa*	39896.40756	Aminoglycosides
	*blaCTX-M-15*	45910.46785	Beta-lactams
	*blaTEM-1B*	49607.50467	Beta-lactams
	*aph(6)-Id*	51188.52024	Aminoglycosides
	*aph(3'')-Ib*	52024.52827	Aminoglycosides
	*sul2*	52888.53703	Sulphonamide
p65L	*blaOXA-48*	27136.27933	Beta-lactams

### Comparative and pan-genomic analysis

The pan-genome and core-genome diversity were analysed with the *Nullarbor* pipeline using the closed Kp11-81 genome as the reference. Core-genome SNV distances to the reference genome showed that all the isolates were closely related except for Kp11-38 that differed from Kp11-81 in >2500 SNVs. The Kp11-38 sequences of the seven genes of the *K. pneumoniae* MLST scheme (*rpoB, gapA, mdh, pgi, phoE, infB, tonB*) were analysed and confirmed that this isolate belonged to ST11. Nevertheless, it was removed from the set.

The remaining 95 isolates had an average of 5247 genes (range 5063–5369) with a core genome of 4514 genes (86 % of the average genome). In addition, 431 genes were present in 95–99 % of the isolates, 359 genes were in 15–95 %, and 864 genes were in <15 %. The core-genome alignment (≈ 4.5 Mb) contained 461 polymorphic positions with 1 to 59 SNVs per isolate (median 6, IQR 4–10) (Fig. S1). Altogether, 286 SNVs were missense variants, 21 introduced premature STOP codons, eighty five were synonymous variants and 69 were in intergenic or non-annotated regions (Table S2). Missense mutations were found in genes from most functional categories though there were several mutations in genes that affect antibiotic susceptibility, e.g. two in *acrR*, two in *acrB*, five in *ompC* (OmpK36), one in *ompF* (OmpK35) (Table S2). The fraction of non-synonymous variants (307) in the polymorphic coding regions of the core genome (392 sites) was 0.78. This value is close to the limit between neutral evolution and positive selection models suggested by Loo *et al*. [28]. This limit is a model-based guideline that must be used carefully, nevertheless, the proximity of the calculated value to the limit suggests a neutral evolution mode, while the mutations affecting antibiotic susceptibility suggest a component of antibiotic driven positive selection.

To analyse insertion and deletion events, the reads from each isolate were mapped to the reference genome. In most isolates the mapping depth of the p220F and p66R plasmids was two to three times that of the chromosome, and the mapping depth of p65L was three to four times that of chromosome. Mapping over the chromosome produced complete coverage with uniform depth for 51 isolates, 39 had deletions ranging from a few base pairs to several kilobases, and five had large duplications (Table S3). Mapping against the p220F plasmid showed full coverage in 16 isolates, 72 had large deletions, one had a large duplication, and the plasmid was not detected in six isolates. The p66R plasmid had full coverage in 33 isolates, large deletions in 48 and was not detected in 12 isolates. Finally, the p65L plasmid had full coverage in 89 isolates, large deletions in six and was not found in one. In this one, Kp11-14, a 25 Kb fragment of the plasmid, that included the *bla_OXA-48_* gene, was detected, but no replicon genes were found. The mapping coverage in this fragment had the same sequencing depth as the chromosome, suggesting that the plasmid had been lost and the fragment had been integrated into the chromosome. Re-sequencing of this isolate showed that indeed, the fragment had been integrated, probably by recombination between a circular DNA fragment containing a retron-type reverse transcriptase gene (*ltrA*) and one of the four chromosomal copies of the same gene (Fig. S3). To study in more detail the parts of the genome of each isolate that were not present in the reference genome, the reads that did not map to the reference were used for *de novo* assembly generating contigs in the range of 1 to 20 Kb. These contigs were analysed by blast against GenBank and by PlasmidFinder. In two closely related isolates (Kp11-53 and Kp11-57) an IncN replicon was detected and in five isolates small Col plasmids were identified (Table S3). In some other isolates contigs that matched plasmid fragments in Genbank were detected, but no replicon could be identified.

Only five isolates showed complete coverage of the reference Kp11-81 genome (chromosome and the three plasmids). The large deletions found in several isolates, and the plasmids and contigs found in some of them and not present in Kp11-81 may account for the non-core genes detected.

### Phylodynamic analysis and dated phylogeny

**Fig. 1.**
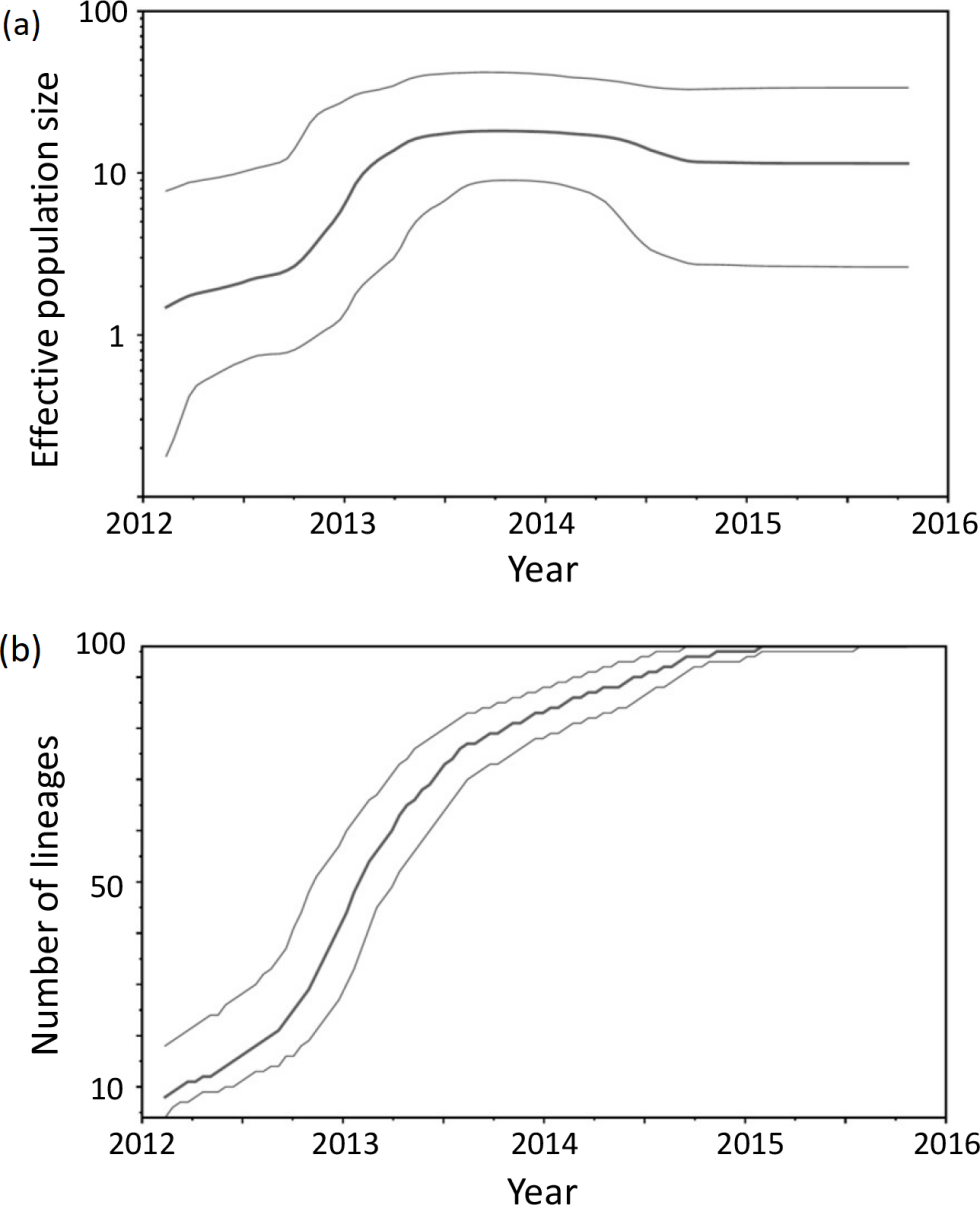
Bayesian inference of the demographic history of our population of ST11 Kp-OXA during the study period. Demography was reconstructed with the Beast 2.6.3 package using a relaxed lognormal clock model and a coalescent bayesian skyline prior. Calculated and drawn with Tracer v1.7.1. (a) Bayesian skyline plot of the effective population size. (b) Number of lineages over time. In both panels, the graphs show the medians and 95 % HPD intervals.

Phylodynamic analyses were done using the core-genome SNVs and the sampling dates. Inference of the demographic history by Bayesian phylodynamic analysis using a relaxed lognormal molecular clock pointed to a rapid and large increase in the effective population size (Fig. 1a) and the number of lineages (Fig. 1b) during the first half of 2013. This is in agreement with a reported increase in the frequency of ST11 Kp-OXA isolates in the same period, when ST11 replaced ST405 as the major type [4].

**Fig. 2.**
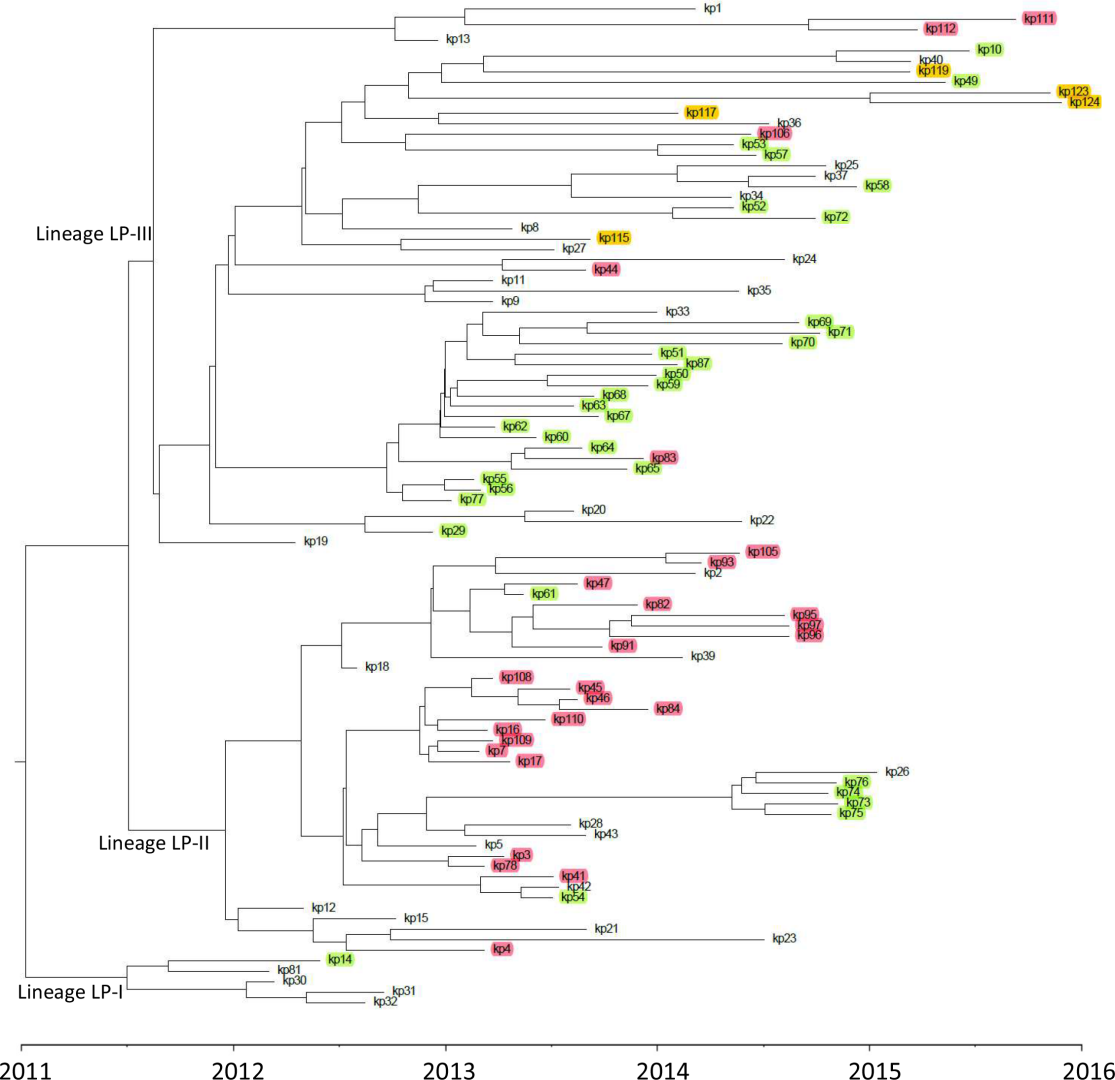
Dated phylogenetic tree of the 96 ST11 Kp-OXA isolates constructed using Bayesian inference with Beast 2.6.3. Trees were summarized as maximum clade credibility tree using TreeAnnotator v1.8.4 and then visualized with FigTree v1.4.4. The three major lineages are indicated. Highlighted: green, Vascular Surgery ward of HULP; magenta, Nephrology ward of HULP; orange, H12O. Not highlighted: isolates obtained from 18 different wards at HULP. Isolate names have been shortened for clarity.

A dated phylogenetic tree was generated using the Beast package (Fig. 2). This was consistent with all 96 isolates descending from a single ancestor dated to December 2010 (95 % HPD: July 2009-January 2012). The sequences were grouped in three lineages, herein named LP-I, LP-II and LP-III, detected for the first time in early 2012. The distribution of isolates in lineages obtained from the core genome SNVs was consistent with the pattern of indels observed (Table S3). A non-Bayesian maximum-likelihood analysis using only SNVs supported lineages LP-I and LP-II with good bootstrap values (85 and 97%, respectively). LP-III, in contrast, was dispersed in several branches with low bootstrap values (Fig. S2). The low resolution of the maximum-likelihood tree is expected in an outbreak setting where the samples are closely related and diversity is limited, highlighting the value of the Bayesian approach including the sample isolation dates.

Lineage LP-I included the reference isolate (Kp11-81). Lineage LP-II included 42 isolates, 21 from the Nephrology ward, six from the Vascular Surgery ward and 14 from other units. Lineage LP-III included 49 isolates, 25 from the Vascular Surgery ward, four from the Nephrology ward, 17 from other wards and the five isolates from H12O. During 2013–2015 there were outbreaks in the Vascular Surgery and Nephrology wards. The tree shows that these were independent events: 21 out of the t26 isolates from Nephrology belonged to lineage LP-II and 25 out of the 32 Vascular Surgery isolates belonged to LP-III. There were also transmission episodes between the two units: five Nephrology isolates were LP-III and six Vascular Surgery isolates were LP-II. Some of these could be associated to patient transfer between units: Kp11-54 and Kp11-61 were LP-II isolates obtained in the surgical ward, yet the two patients were Nephrology patients; on the other hand, Kp11-83 and Kp11-106 were LP-III isolates obtained in the medical ward, yet Kp11-83 was obtained from a patient admitted to Vascular Surgery ten days before the sample was taken, and Kp11-106 was isolated from a patient admitted from a long-term care facility 3 days before. Aside from these two units, in up to four different units with more than one isolate sequenced two or the three lineages were detected (Table S3).

The dated phylogeny showed also that some lineages persisted for several months in the same unit (e.g. the Kp11-30, Kp11-31 and Kp11-32 isolates were obtained in a paediatric unit over a period of 6 months), and that some apparent transmission clusters were actually independent events (e.g. isolates Kp11-82, Kp11-83 and Kp11-84 were obtained between 31 October and 18 November 2013 in the Nephrology ward, but Kp11-83 belonged to LP-III and Kp11-82 and Kp11-84 belonged to two different branches within LP-II).

The five isolates from H12O belonged to LP-III and had a common ancestor with other LP-III isolates dating back to early 2012, although ST11 Kp-OXA were not detected in H12O until 2013 [29].

## Discussion

Previous studies had shown that the Kp-OXA epidemic is polyclonal but dominated by a few *K. pneumoniae* major lineages [3, 30]. One of these was ST11, a lineage with worldwide distribution and known to cause hospital outbreaks of ESBL- and carbapenemase-producers. With such a broad distribution ST11 has a large and diverse population [5, 31] yet the isolates from the same hospitals tend to be more related between them than to those of other sites [31] showing a pattern of local expansion. To understand the evolution of a large Kp-OXA hospital epidemic we have sequenced and analysed a collection of 97 ST11 Kp-OXA isolates obtained from two hospitals in Madrid during the early years of the outbreak. We found that 96 of them were closely related, most having less than 20 SNVs when compared to the reference sequence. This suggested that they might be part of a single transmission cluster with a recent common ancestor. The moderate pan-genome diversity found was due for the most part to large deletions and plasmid acquisitions. The large size of the core genome compared to the average genome size (86%) supports the close relationship between the isolates. Different cutoff values for the number of core genome SNVs have been proposed to define transmission clusters, ranging from 11 [32] to 21 [33] or even 300 [34]. In our data a cutoff of 21 SNVs would cluster 87 isolates, yet the remaining nine might be included in the same group on the basis of coverage, shared SNVs and indels. The dated phylogeny inferred by the Bayesian approach suggested that all ninety six isolates had a common ancestor that had acquired the p65L plasmid towards the end of 2010.

One isolate, Kp11-38, was excluded from the analysis because it was very distant from all the others in spite that it belonged to ST11 and must have acquired the plasmid harbouring the OXA-48 gene independently of the major lineage. All the remaining isolates could be grouped into a single transmission cluster. This is consistent with an epidemic structure driven by few (high-risk) Kp-OXA lineages that become dominant over a background of low frequency and sporadic isolates belonging to other lineages and species and resulting from multiple within-host horizontal gene-transfer events [27, 35–37].

During the first years of the outbreak, using DNA fingerprinting (Diversilab) and MLST, we described a mixed pattern with isolates of one ST detected in different hospital wards and isolates from different STs found in the same hospital ward [3, 38, 39]. Delving deeper into the genome sequences increases the resolution and generates a more detailed image that confirms the pattern of local (ward level) lineage expansion and occasional transmission between different hospital units in HULP, and even between different hospitals. Community transmission of Kp-OXA is uncommon [27], suggesting that HULP is the most likely source of the Kp-OXA of H12O, either directly by patient transfer or indirectly through an intermediate healthcare institution. This pattern is likely the result of the ability of Kp-OXA to silently colonize the human intestine, reaching very high bacterial loads that may persist for a long time in hospitalized patients [36, 40, 41]. Patients with high Kp-OXA intestinal loads are likely to be major sources driving the spread to other patients, either directly, through hospital staff or through shared devices. They also contaminate their immediate environment [42] that may become a long-term reservoir [27, 43]. The median age of Kp-OXA colonized patients in HULP during the study period was around 70 years [3, 30], many of the patients had co-morbidities that often required patient transfer between wards or recurrent hospitalizations in different hospital wards, driving or facilitating the dissemination of Kp-OXA through the hospital. The number of samples sequenced was small compared to the number of positive samples obtained [30], they provided a general view of ST11 genomic diversity during the epidemic but no attempt was done to correlate genotypes with patient movement or transmission events outside the Vascular Surgery and Nephrology wards. The study was focused on these two units because both have their own patients but they are often involved in patient transfer to and from other wards and the two units had simultaneous outbreaks of ST11 Kp-OXA in early 2013. Genomic analysis showed that these were independent outbreaks produced by different ST11 lineages, LP-II in Nephrology and LP-III in Vascular Surgery, and that transmission occurred within and between wards, between hospitals, and between care facilities and hospitals. This underlines the importance of active high resolution (i.e. genomic) surveillance programmes [44, 45] because in the context of a large hospital-wide epidemic, neither the outbreaks nor the transmission between wards can be correctly differentiated with lower resolution typing methods (such as MLST or PFGE). Genome sequencing has already become fast and cheap enough to be used extensively for rapid epidemiological surveillance, tracking of transmission pathways and guiding infection control.

## Supplementary Data

Supplementary material 1

Supplementary material 2
