# Supplementary material for: Dissemination of a single ST11 clone of OXA-48-producing Klebsiella pneumoniae within a large polyclonal hospital outbreak determined by genomic sequencing
Source: Microb Genom. 2022 Apr 8;8(4):000808. doi: 10.1099/mgen.0.000808 (PMC9453077; doi:10.1099/mgen.0.000808)
Supplement: Supplementary material 2 [file mgen-8-0808-s001.pdf]

Supplementary Files

Supp. Table 1. Summary of the sequencing data. The raw reads have been deposited in the Genbank database under Bioproject accession number PRJNA757198.

Supp. Table 2. Description of the 461 core genome polymorphic positions and SNVs found in each isolate. In columns Kp11-1 to Kp11-124, dots indicate positions identical to the reference sequence Kp11-81.

Supp. Table 3. Summary of the sequence microevolution. For each isolate, the table shows the isolation date and ward codes, the lineage, number of SNVs, indels and presence/absence of plasmids. Code G02C is the Vascular Surgery ward, code G08C is the Nephrology ward and code H12O is Hospital Doce de Octubre. The coordinates of indels are given in Kb in reference to the Kp11-81 sequence.

Supp. Fig. 1. Plot of the distribution of SNVs as a function of time from the reference Kp11-81 isolate. Each point represents one isolate. The isolates with the highest SNV numbers are labeled and closely related isolates encircled. The dotted lines mark the SNV cutoff values proposed in refs. 32 and 33 to define transmission clusters. The number of SNVs per isolate follows a lognormal distribution (Kolmogorov-Smirnov test of normality for log(number of SNVs), p-value=0.29).

Supp. Fig. 2. Consensus phylogeny of the ninety six ST11 Kp-OXA isolates obtained by maximum likelihood method using IQtree (http://iqtree.cibiv.univie.ac.at) [1, 2]. Evolutionary model selection was done with ModelFinder, trees were constructed with IQ-TREE and bootstrap values calculated with UFBoot [3]. The consensus was obtained from 1000 bootstrap trees. The tree is unrooted although taxon 'kp1' is drawn at root. Drawn with FigTree v1.4.4.

Supp. Fig. 3. Comparison of the genetic organization of the chromosomal region containing the *bla*$_{OXA-48}$ gene in Kp11-14 and the ancestral regions in Kp11-81. The genetic structures suggest that a fragment of the p65L plasmid containing the *bla*$_{OXA-48}$ gene (yellow) was excised and formed an intermediary circular DNA fragment by homologous recombination between two IS1 elements (magenta), this intermediate was then inserted into a chromosomal copy of the *ltrA* retron-type reverse transcriptase gene (red). The reference chromosomal sequence is colored in cyan, same as the equivalent sequence in Kp11-14. The two segments of the p65L plasmid sequence are colored in green and blue.

References

1. **Kalyaanamoorthy S, Minh BQ, Wong TKF, von Haeseler A, Jermiin LS**. ModelFinder: fast model selection for accurate phylogenetic estimates. *Nat Methods* 2017;14:587–589.

2. **Nguyen L-T, Schmidt HA, von Haeseler A, Minh BQ**. IQ-TREE: A Fast and Effective Stochastic Algorithm for Estimating Maximum-Likelihood Phylogenies. *Mol Biol Evol* 2015;32:268–274.

3. **Hoang DT, Chernomor O, von Haeseler A, Minh BQ, Vinh LS**. UFBoot2: Improving the Ultrafast Bootstrap Approximation. *Mol Biol Evol* 2018;35:518–522.

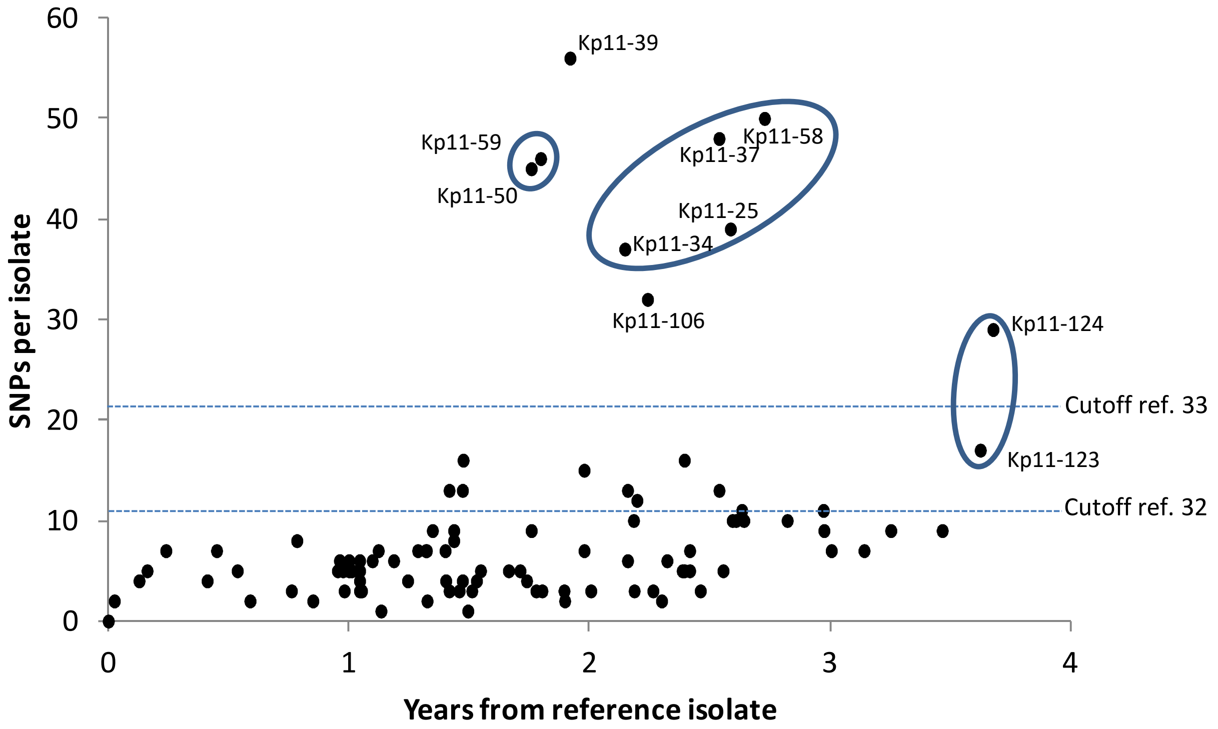

Supp. Fig. 1. Distribution of the number SNVs of the 96 isolates as a function of time from the reference Kp11-81 isolate. Each point represents one isolate. The isolates with the highest SNV numbers are labeled and closely related isolates encircled. The dotted lines mark the SNV cutoff values proposed in refs. 32 and 33 of the main text to define transmission clusters. The number of SNVs per isolate follows a lognormal distribution (Kolmogorov-Smirnov test of normality for log(number of SNVs), p-value=0.29).

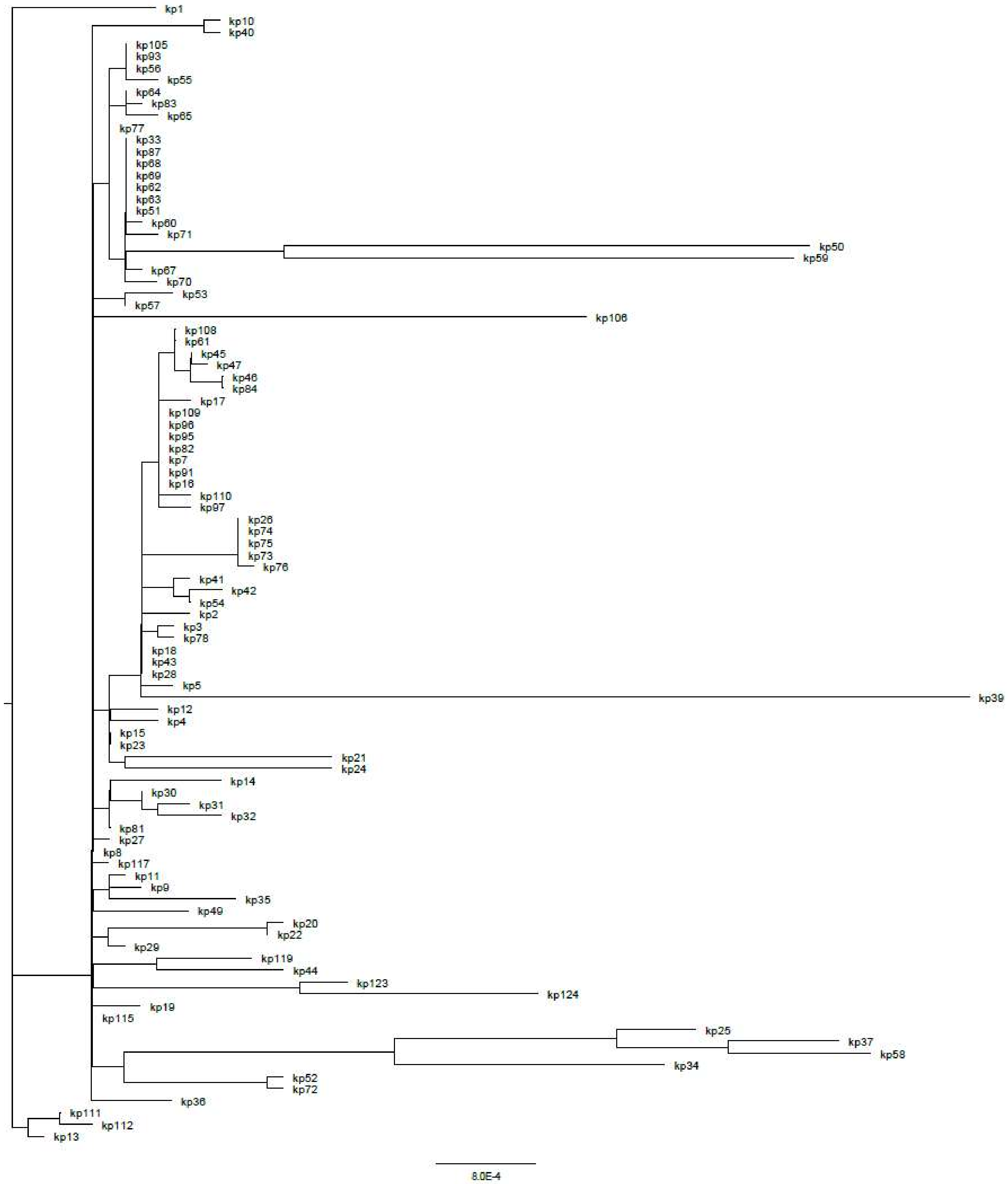

Supp. Fig. 2. Consensus phylogeny of the ninety six ST11 Kp-OXA isolates obtained by maximum likelihood method using IQtree (http://iqtree.cibiv.univie.ac.at) [1, 2]. Evolutionary model selection was done with ModelFinder, trees were constructed with IQ-TREE and bootstrap values calculated with UFBoot [3]. The consensus was obtained from 1000 bootstrap trees. The scale is in nucleotide substitutions per nucleotide site (calculated for 461 polymorphic sites with ascertainment bias correction). The tree was drawn with FigTree v1.4.4.

1. **Kalyaanamoorthy S, *et al***. ModelFinder: fast model selection for accurate phylogenetic estimates. *Nat Methods* 2017;14:587–589.
2. **Nguyen L-T, *et al***. IQ-TREE: A Fast and Effective Stochastic Algorithm for Estimating Maximum-Likelihood Phylogenies. *Mol Biol Evol* 2015;32:268–274.
3. **Hoang DT, *et al***. UFBoot2: Improving the Ultrafast Bootstrap Approximation. *Mol Biol Evol* 2018;35:518–522.

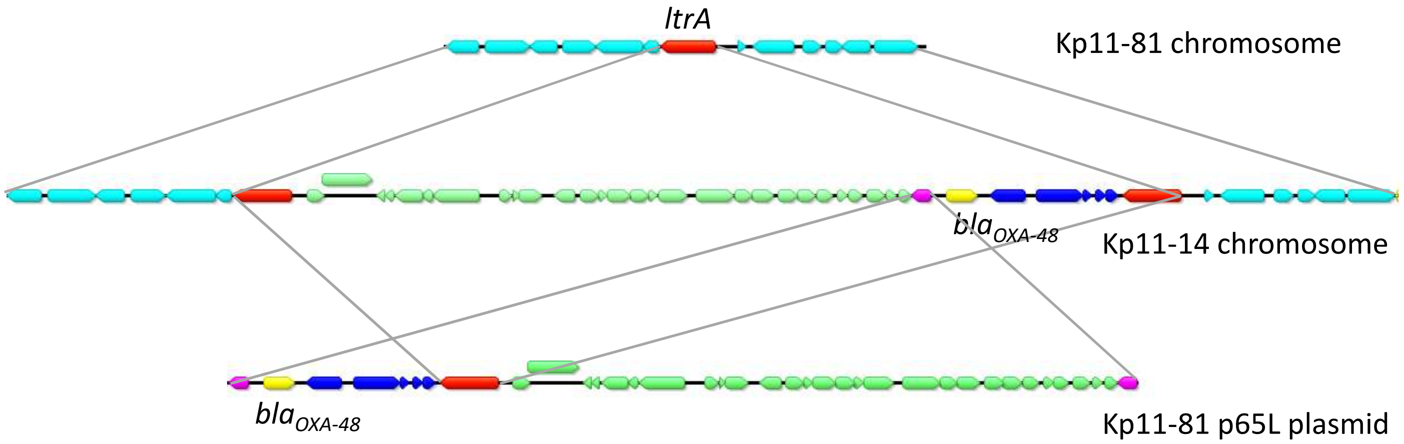

Supp. Fig. 3. Comparison of the genetic organization of the chromosomal region containing the *bla*$_{OXA-48}$ gene in Kp11-14 and the ancestral regions in Kp11-81. The genetic structures suggest that a fragment of the p65L plasmid containing the *bla*$_{OXA-48}$ gene (yellow) was excised and formed an intermediary circular DNA fragment by homologous recombination between two copies of an IS1 element (magenta), this intermediate was then inserted, again by homologous recombination, into one of the four chromosomal copies of the *ltrA* retron-type reverse transcriptase gene (red). The reference chromosomal sequence and the equivalent sequences in Kp11-14 are colored cyan. The fragment of the p65L plasmid is colored in green and blue to show the change in the relative order of the two segments surrounding the *ltrA* gene.
